# Traditional Chinese acupoint massage, acupuncture, and moxibustion for people with diabetic gastroparesis: A systematic review and meta-analysis

**DOI:** 10.1097/MD.0000000000032058

**Published:** 2022-12-02

**Authors:** Xiaoming Li, Zongbao Yan, Jin Xia, Yanan Sun, Peijun Gong, Yuncui Fan, Xiaodong Wang, Xinjie Cui

**Affiliations:** a The Third Clinical Medical College, Zhejiang Chinese Medical University, Hangzhou, China; b College of Basic Medical Science, Zhejiang Chinese Medical University, Hangzhou, China.

**Keywords:** acupoint, acupuncture, diabetic gastroparesis, massage, meta-analysis, moxibustion, Traditional Chinese Medicine

## Abstract

Traditional Chinese acupoint therapy has been used for thousands of years on gastrointestinal diseases. In this work, we evaluated the efficiency and safety of traditional Chinese acupoint therapies versus standard therapies, nursing or recovery treatments in the treatment of diabetic gastroparesis.e expect that traditional Chinese medicine acupoint therapy can be noticed by more people, so as to provide more high-quality clinical evidence.

**Methods:**

Randomized controlled trials were included in this meta-analysis. The treatment groups received traditional Chinese acupoint therapy, while the control groups received standard therapies, nursing, or recovery treatments. The relative risk and weighted mean difference with 95% confidence interval for the total effective rate, gastrin level, gastric-emptying time, fasting blood glucose level, 2-hour blood glucose level, and glycosylated hemoglobin level were evaluated using RevMan 5.3 software. Bias assessment was performed using the Cochrane risk-of-bias tool.

**Results:**

A total of 59 articles were included in the analysis. In comparison with the control groups, the acupoint therapy groups showed higher total effective rates (*P* < .00001), enhanced gastric-emptying rates (*P* < .00001), and reduced glycosylated hemoglobin levels.

**Conclusion:**

In comparison with Western medicine or conventional care, traditional Chinese acupoint therapies showed a significant advantage in the treatment of diabetic gastroparesis. However, considering the low quality and high risk of the included studies, more high-quality randomized controlled trials are needed to confirm the results.

## 1. Introduction

Gastroparesis is defined as a delay in the emptying of ingested food in the absence of mechanical obstruction of the stomach or duodenum.^[1]^ Symptoms of gastroparesis include nausea, vomiting, early satiety, postprandial fullness, bloating, and upper abdominal pain.^[2]^ Gastroparesis is a common chronic complication of diabetes mellitus. Since most patients with gastroparesis may show atypical or no symptoms, the disease is easily ignored by patients and clinicians. Insulin is typically used to control hyperglycemia in patients with diabetes. However, insulin also delays gastric emptying. Therefore, the long-term use of insulin to control blood sugar may weaken the gastric-emptying function of patients with diabetes, aggravating the symptoms of gastroparesis.^[3]^

The channel theory and viscera-bowels theory are core principles in Traditional Chinese Medicine (TCM).^[4]^ The viscera, the main organ of the human body, connects with the whole body through meridians. According to classical acupuncture theory, disorders of visceral conditions and organs are reflected at specific points, either on the skin surface or underneath, and these points are generally called acupoints.^[5]^ Acupoint therapies such as acupuncture, massage, and moxibustion modify the operation of the meridians by acting on the acupoints to adjust Qi-blood circulation of the body and treat diseases. Acupoint therapy has been proven to have a specific curative effect and shows notable advantages in the treatment of diabetes and its common chronic complications.^[6]^ This study systematically reviewed clinical randomized controlled trials (RCTs) of acupoint therapies for the treatment of type 2 diabetic gastroparesis with the aim of investigating the efficacy and safety of acupoint therapy and providing supporting evidence.

## 2. Materials and Methods

The systematic review and meta-analysis was registered in PROSPERO (registration number: CRD42020179387). No protocol was used for this meta-analysis. The ethical approval was not necessary and waived.

### 2.1. Search strategy

The search strategy was determined according to the guidelines of the PRISMA agreement.^[7]^ RCTs in humans were also included. Articles in English and Chinese were searched in 5 databases, namely, PubMed, Embase, Cochrane Library, Chinese National Knowledge Infrastructure, and Wanfang Data Information Site, from inception to 04/01/2020. Medical subject headings (MeSH) terms and keywords were used to retrieve related studies. The search terms were (“DGP” or “diabetic gastroparesis” or “diabetic gaetmparcais” or “Diabetogenous gastroparesis”) and (“massage” or “massotherapy” or “Tuina” or “akupunktur” or “acupuncture” or “needling” or “electropuncture” or “moxa-moxibustion” or “moxibustion”).

### 2.2. Study selection

The trials included in this meta-analysis evaluated patients diagnosed with diabetic gastroparesis on the basis of symptoms and abnormal physiological test results. The main symptoms included hiccups, upper abdominal fullness, anorexia, nausea, vomiting, and other gastrointestinal symptoms. Electrogastric examination revealed abnormal gastric motor function, and a scintillation scan of gastric emptying showed delayed gastric emptying. Participants with other types of gastroparesis were excluded. The inclusion criteria were as follows:

1. Control groups received standard therapies, nursing, or recovery treatments, including conventional Western medicine treatments such as gastric motility drugs, hypoglycemic drugs, and health education. In addition, the comparators included no treatment, placebo, or other treatments.
2. Treatment groups received interventions such as acupuncture, massage, or moxibustion alone or together, or in combination with standard therapies, nursing, or recovery treatments.
3. RCTs were included.
4. The main outcomes included the gastric-emptying rate, gastric dynamic element, gastric secretion element, and effective rate. The effective rate is the sum of “effective rate” and “ markedly effective rate.” Additional outcomes included quality of life score, fasting blood glucose (FBG), 2-hour postprandial blood glucose, and glycated hemoglobin level (HbA1c).
5. Trials in which the control or treatment group received oral Chinese medicine were excluded.

### 2.3. Data extraction

Two investigators (Xinjie Cui and Jin Xia) searched the databases listed above independently, downloaded all articles that met the inclusion criteria, and eliminated duplicate articles. Two reviewers (Xiaoming Li and Zongbao Yan) independently reviewed the articles and determined their suitability for inclusion in the study. Three other reviewers (Yanan Sun, Peijun Gong, and Yuncui Fan) independently extracted data from each study. When the reviewers had different opinions on an article, an additional investigator (Xiaodong Wang) performed an independent review to determine whether the study should be included. The data extracted from the articles were as follows: name of the first author, year of publication, number of intervention and control groups, interventions and courses in the intervention and control groups, acupoints, and the main outcomes.

### 2.4. Quality assessment

The risk of bias was assessed for all included articles using Cochrane Collaboration RevMan software (version 5.3). The assessed elements included random sequence generation, allocation concealment, blinding of outcome assessors, incomplete outcome data, selective outcome reporting, and other sources of bias. Disagreements were resolved through discussion with a third reviewer (Xiaodong Wang).

### 2.5. Statistical analysis

Statistical analyses were conducted using RevMan (Version 5.3; The Cochrane Collaboration, Copenhagen, The Nordic Cochrane Centre). Data types included dichotomous and continuous data. For dichotomous data, such as the total effective rate, the risk ratio (RR) with a 95% confidence interval (CI) was used to combine the data from the trials. For continuous data such as gastrin secretion, gastric-emptying time, fasting blood glucose, 2-hour blood glucose, and glycosylated hemoglobin, the weighted mean difference with 95% CI was used to combine data from the trials. When different measurement scales were used, we used the standardized mean difference to combine the continuous data from the trials. The *I*^2^ test was used for heterogeneity analysis. Heterogeneity was defined by an *I*^2^ value ≥50%. We used a random effects model and conducted a sensitivity or subgroup analysis to analyze heterogeneity. The literature exclusion method was used for sensitivity analysis. When the *I*^2^ value was less than 50%, which represented homogeneity, a fixed-effect model was used. Publication bias was analyzed using a funnel plot analysis if sufficient studies (n > 10) were identified, and values of *P* < .05 were considered statistically significant.

## 3. Results

### 3.1. Study selection

**Figure 1.**
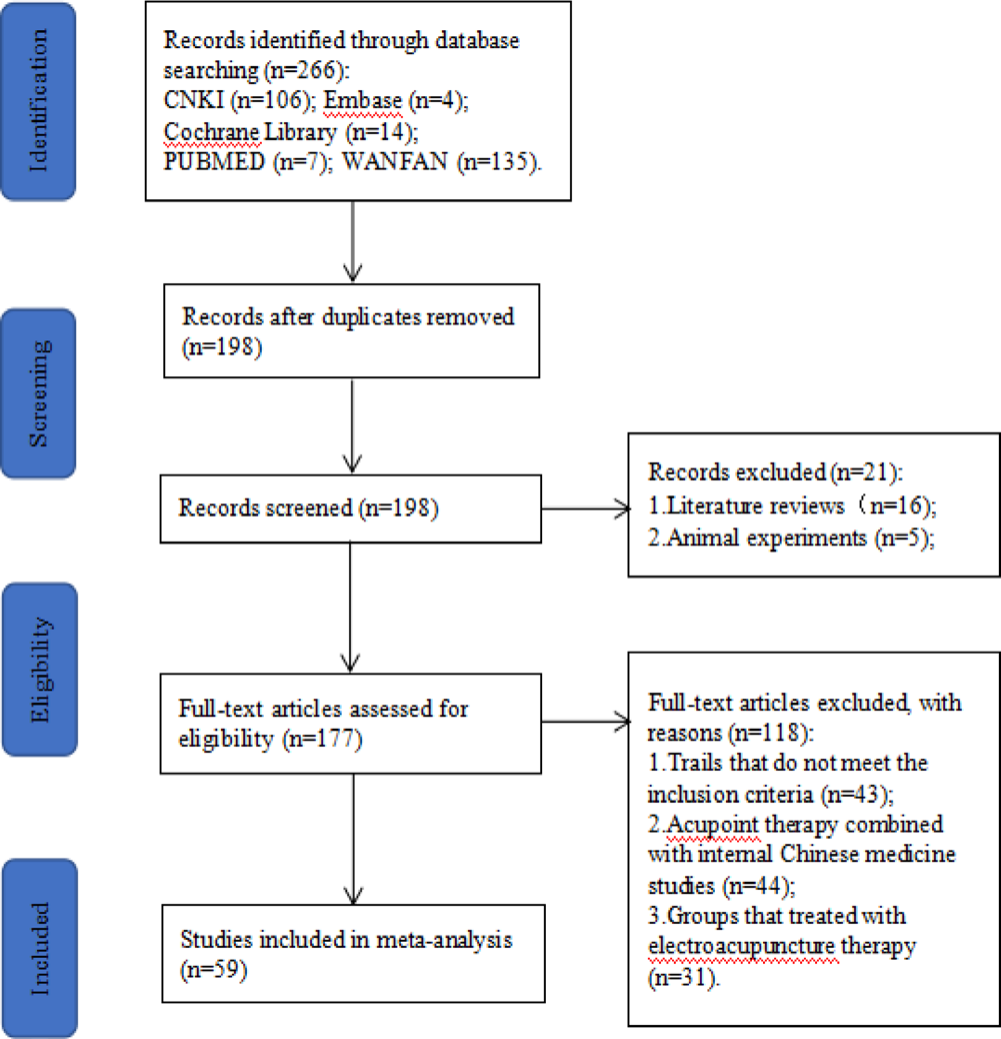
The flow diagram of the study selection process.

We searched 266 articles from the 5 databases and excluded 68 duplicate articles using NoteExpress 3.2.0.7535. We read the titles and abstracts and further excluded 21 reviews and animal experiments. We then downloaded the remaining 177 articles and reviewed them to exclude those that did not meet the inclusion criteria; the excluded articles mainly included non-randomized controlled trial studies, acupoint therapy combined with internal Chinese medicine studies, and non-type 2 diabetic gastroparesis studies. Finally, 59 articles were included in the meta-analysis. Figure 1 is the summary of the study selection process.

### 3.2. Characteristics of the included studies

A total of 59 RCTs were included in this meta-analysis, and the detailed characteristics of the included trials, such as treatment measures, course of treatment, and acupoints used, are listed in Table 1. In these studies, the treatment groups received one or more acupoint therapies combined with conventional care or Western medicine, and the control group received conventional care alone or combined with Western medicine. The 59 studies included 10^[25,29,30,34,38,44,52,54,61,63]^ studies using massage therapy, 22^[8,12,14–16,20,22,26,28,35,43,47,49,50,53,55,57–60,64,65]^ using acupuncture therapy, 8^[11,18,31,33,36,48,56,66]^ using moxibustion therapy, and 19^[9,10,13,17,19,21,23,24,27,32,37,39–42,45,46,51,62]^ using combined acupoint therapies. Point percussion, chiropractic, and auricular acupressure therapies were considered massage therapies. As for the outcomes, 53 studies reported the total effective rate, of which 42^[8–11,14,15,17–25,27,28,33–35,37–40,43–45,47–49,51,53–61,63,66]^ were based on indicators of Western medicine and 13^[12,13,16,17,29–31,36,50,52,53,62,65]^ were based on TCM symptoms. Fifteen studies analyzed the gastric-emptying function, of which 5^[37,38,41,47,63]^ evaluated the gastric-emptying time, 4^[33,40,42,48]^ assessed the gastric-emptying rate, and 6^[13–15,24,44,62]^ assessed the gastric barium bar content after a certain period of time. In addition, 16 studies^[11,21,23,29,30,34,37,38,43–45,47–49,52,64]^ reported FBG levels, of which 9^[23,29,30,34,47–49,52,64]^ reported 2-hour blood glucose, 7^[11,26,29,44,45,48,55]^ reported glycosylated hemoglobin levels, 4^[13,15,23,33]^ reported gastrin levels, and 6^[13,15,23,33,38,55]^ reported the presence of motilin.

**Table 1:** Detailed characteristics of the included trials.

First author (year)	Sample (T/C)	Age (T/C, yr)	Intervention (E/C)	Course of the treatment	Acupoints	Main outcomes
Bai (2001)^[8]^	65 (35/30)	55.1 ± 15.4/54.2 ± 16.3	T4, T7/T7	2 per D for T4 and 3 per D for T7/3 per D (2W)	A18,A16	E1
Cheng (2003)^[9]^	72 (36/36)	53 ± 12/51 ± 15	T2, T3, T4/T2, T8, T14	1 per D/3 per D for T8 and T14 (1M)	A16, A1, A13, A40, A22, A41, A12, A20, A42, A15, A43, A44	E1, E5, E12
Deng (2012)^[10]^	50 (25/25)	31–76/36–75	T1, T3, T6/T1, T9	1 per D/3 per D (1M)	T3: A2; T6: A13, A20, A23	E1, E2
Fu (2011)^[11]^	60 (30/30)	47–74/45–69	T1, T2, T3/T1, T2, T8	1 per D/3 per D (10D)	A20, A13, A23, A1, A40, A19, A15	E1, E5, E6
Ge (2010)^[12]^	60 (30/30)	40–61/42–60	T1, T2, T4/T1, T2, T8	1 per D/3 per D (1M)	A16, A8, A1	E2
Ge (2016)^[13]^	100 (50/50)	57.1 ± 3.7/58.5 ± 4.6	T1, T2, T6/T1, T2, T8	1 per D/3 per D (1M)	A1, A16, A8	E2, E3, E11
Ge (2015)^[14]^	80 (40/40)	63.9 ± 5.7/64.2 ± 5.3	T2, T4/T2, T8	1 per D/3 per D (1M)	A1, A16, A8	E1, E2, E3
Ge (2016)^[15]^	100 (50/50)	58.0 ± 7.5/58.5 ± 5.7	T1, T2, T4/T1, T2, T8	1 per D/3 per D (1M)	A1, A16, A8	E1, E2, E3, E11
Guo (2018)^[16]^	60 (30/30)	–	T4/T9	1 per D/3 per D (1M)	A24, A25	E2
Guo (2010)^[17]^	60 (30/30)	33–66/32–67	T2, T3, T4/T2, T7	1 per D/3 per D (2W)	T3: A2; T4: A1, A16, A46, A15	E1, E2
Han (2017)^[18]^	64 (32/32)	47.4 ± 3.6/48.1 ± 3.2	T1, T2, T3/T1, T2, T8	1 per D/3 per D (1M)	A16	E1, E2
He (2017)^[19]^	60 (30/30)	43.1 ± 3.5/48.5 ± 2.6	T1, T3, T9, T10/T1, T9	1 per D for T3; 3 per D for T9 and T10/3 per D (1M)	T3: A8, A16, A1	E1, E2, E3
Jin (2014)^[20]^	70 (35/35)	40–75	T4/T8	1 per D (2W)	A1, A16, A8, A17, A18	E1
Jin (2016)^[21]^	59 (31/28)	55.81 ± 9.24/54.00 ± 8.51	T2, T6/T2	1 per D (15D)	A1, A16, A8, A15, A19	E1, E5
Kong (2009)^[22]^	90 (45/45)	66.93 ± 7.77/65.07 ± 8.05	T1, T2, T4/T1, T2, T7	1 per D/3 per D (1M)	A1, A8, A18, A16, A17	E1
Li (2017)^[23]^	66 (33/33)	49.85 ± 3.21/9.24 ± 1.39	T2, T4, T8, T3/T2, T8	1 per D for T3 and T4; 3 per D for T2/3 per D (28D)	A8, A16, A21, A1,A13, A15, A20, A23	E1, E5, E10, E11
Li (2015)^[24]^	100 (50/50)	45.1 ± 9.9/45.0 ± 10.5	T6/T8	1 per D (20D) /3 per D (1M)	A1, A16, A15	E1,E3
Li (2015)^[25]^	100 (50/50)	–	T1, T2, T5/T1, T2, T8	1 per D/3 per D (1M)	A47, A13, A20, A42, A48, A22, A16, A49, A50, A17, A1, A2, A18	E1
Liu (2012)^[26]^	70 (35/35)	47.3 ± 8.71/49.1 ± 9.37	T2, T4, T9/T2, T9	1 per D (1M)	A1, A13, A14, A15, A16	E6, E7, E8
Liu (2001)^[27]^	44 (25/19)	52–74/50–73	T1, T2, T3, T4, T8/T1, T2, T8	1 per D for T3 and T4; 3 per D for T8/3 per D (1M)	T3: A41, A12; T4: A56, A8, A1, A16	E1
Liu (2010)^[28]^	90 (45/45)	59 ± 10	T4/T8	1 per D/3 per D (10D)	A16, A8, A1, A20, A13, A11, A12, A17	E1
Liu (2012)^[26]^	60 (30/30)	–	T1, T3, T13/T1, T13	5–7 per D for T3; 3 per D for T13/3 per D (1M)	A16, A1, A8	E1
Long (2018)^[29]^	60 (30/30)	50–70	T1, T5, T8, T12/T1, T8	–	A12, A1, A18, A11	E2, E5, E6, E10
Lu (2006)^[30]^	106 (53/53)	45 ± 18/45 ± 20	T1, T2, T5, T11/T1, T2, T9	2 per D/3 per D (1M)	–	E2, E5, E10
Mai (2016)^[31]^	70 (35/35)	–	T1, T2, T8, T3/T1, T2, T8	1 per D for T3; 3 per D for T8/3 per D (10D)	A2, A1, A16	E2
Mao (2009)^[32]^	59 (30/29)	42–76/43–76	T2, T3, T4/T2, T9	1 per D/3 per D (1M)	T4: A45, A52, A8, A24, A26, A44, A31, A13; T3: A1, A12, A16	E2
Meng (2020)^[33]^	134 (67/67)	46 ± 10/47 ± 11	T3/T13	1 per D/3 per D (42D)	A39	E1, E2, E11, E13
Min (2019)^[34]^	60 (30/30)	61.6 ± 7.55/61.57 ± 6.73	T1, T2, T10, T11/T1, T2, T9	1 per D for T10; 1 per 2D for T11/3 per D (1M)	A45, A42, A22, A13, A20	E1, E2, E5, E10
Mo (2006)^[35]^	81 (41/40)	51.7 ± 5.5/55.9 ± 7.1	T1, T4/T1, T8	1 per D/3 per D (28D)	A8, A1, A16, A17	E1
Ruan (2014)^[36]^	100 (50/50)	58.8 ± 16.2/55.2 ± 14.8	T1, T2, T3/T1, T2	1 per D (1M)	A1, A2	E2
Shen (2010)^[37]^	60 (30/30)	52 ± 10.5/50 ± 10.2	T2, T4, T15/T2, T8	1 per D/3 per D (1M)	T4: A8, A1, A11, A16, A15; T15: A48, A13, A20, A18	E1, E2, E4, E5
Song (2004)^[38]^	100 (50/50)	54.2 ± 5.7/53.9 ± 5.7	T1, T2, T5, T8/T1, T2, T8	1 per D for T4; 3 per D for T8/3 per D (42D)	A1, A16	E1, E2, E4, E5, E11
Sun (2007)^[39]^	66 (35/31)	67/61	T1, T3, T10/T1, T7	1 per D/3 per D (1M)	–	E1
Sun (2018)^[40]^	104 (52/52)	64 ± 6/66 ± 5	T2, T4, T16/T2, T8	1 per D for T4; 3 per D for T16/3 per D (2M)	A5, A51, A35	E1, E2, E13
Wang (2009)^[41]^	70 (35/35)	26–65/28–69	T1, T2, T4, T6/T1, T2, T7	1 per D (1M)	A9, A10, A1, A4, A2, A11, A12	E3, E4, E5, E6
Wang (2014)^[42]^	64 (31/33)	56.91 ± 4.27/55.52 ± 4.68	T1, T2, T4, T10/T1, T2, T9	1 per D for T4; 3–5 per D for T10/3 per D (24D)	T4: A8, A1, A16, A17	E2, E9
Wang (2009)^[43]^	76 (40/36)	–	T2, T4/T2, T8	1 per D/3 per D (1M)	A1, A16, A15, A53, A24, A54	E1, E2, E5
Wang (2009)^[44]^	106 (53/53)	45 ± 18/45 ± 20	T1, T2, T5, T11/T1, T2, T9	1 per D/3 per D (1M)	–	E1, E5, E10
Wang (2010)^[45]^	70 (35/35)	37–86/40–85	T1, T2, T4, T11/T1, T2, T9	1 per D/3 per D (1M)	A8, A16, A15, A1	E1, E2, E5, E6
Wang (2012)^[46]^	72 (36/36)	42–68/45–70	T1, T4, T6, T7/T1, T7	1 per D for T4; 3 per D for T7/3 per D (20D)	T6: A1, A16; T4: A8, A15, A24, A20, A31, A13, A19, A22	E1
Wang (2018)^[47]^	128 (64/64)	63.37 ± 6.92/63.58 ± 7.01	T4, T9/T9	1 per D/3 per D (1M)	A16, A12, A14, A30, A24, A32, A15, A26, A31, A14, A19 A33	E1, E2, E4, E5, E6
Wang (2018)^[48]^	90 (45/45)	36.28 ± 5.71/37.64 ± 5.92	T1, T2, T3, T13, T14/T1, T2, T13, T14	1 per D for T4; 3 per D for T13,T14,10/3 per D (10D)	A16, A1, A18, A2	E1, E2, E5, E10, E13
Wang (2019)^[49]^	128 (64/64)	56.2 ± 2.2/53.4 ± 2.1	T4, T9/T9	1 per D for T4; 3 per D for T9/3 per D (1M)	A16, A12, A14, A30, A24, A32, A15, A26, A31, A19, A33	E1, E2, E5, E10, E12
Wu (2015)^[50]^	120 (60/60)	52 ± 11/54 ± 10	T4, T8/T8	1 per D (1M)	A3, A1, A4, A5, A6, A7, A8	E2
Wu (2008)^[51]^	70 (35/35)	26–65/28–69	T1, T4, T6/T1, T7	1 per D/3 per D (1M)	T4: A9, A10, A1, A4, A11, A12; T6: A11, A12, A2	E1
Xu (2015)^[52]^	84 (42/42)	61 ± 8/59 ± 8	T1, T2, T5/T1, T2, T9	1 per D/3 per D (22D)	A1, A18, A16, A8	E2, E5, E10
Xu (2016)^[53]^	82 (41/41)	35–75	T1, T2, T4/T1, T2	1 per D/3 per D (45D)	A1, A8, A16, A18, A15, A19, A13, A20	E1, E2
Xu (2012)^[54]^	64 (32/32)	60–75/60–76	T1, T2, T10, T12/T1, T2, T8	2 per D/3 per D (1M)	A1, A18, A16, A8	E1
Yang (2013)^[55]^	70 (35/35)	37–86/34–85	T1, T4/T1, T9	1 per D/3 per D (1M)	A1, A4, A11, A12, A18, A34, A35, A36, A37, A38	E1, E2,E6, E11
Yu (2016)^[56]^	80 (40/40)	59.2 ± 5.2/58.2 ± 5.1	T1, T3/T1, T8	1 per D, then 1 per 2D/3 per D (2W)	A16	E1
Zeng (2008)^[57]^	60 (30/30)	53 ± 12/51 ± 15	T1, T2, T4/T1, T2, T8	1 per D/3 per D (1M)	A1, A16, A8, A15	E1, E2
Zhang (2009)^[58]^	45 (26/19)	46–70	T4/T8	1 per D/3 per D (21D)	A16, A8, A1	E1
Zhang (2013)^[59]^	194 (98/96)	47.62 ± 5.23/46.96 ± 6.12	T4/T8	1 per D/3 per D (21D)	A16, A8, A1	E1, E2
Zhang (2007)^[60]^	72 (36/36)	47.26 ± 5.13/48.31 ± 6.57	T1, T2, T4/T1, T2, T8	2 per D/3 per D (1M)	A14, A25, A1, A16, A21, A26, A15, A27, A28, A24, A17, A29, A18	E1, E2
Zhang (2009)^[61]^	78 (40/38)	58.1 ± 6.1	T10/T2, T8	3–4 per D/3 per D (1M)	–	E1
Zheng (2010)^[62]^	80 (40/40)	44.7 ± 8.9/43.9 ± 9.1	T6/T8	1 per D/3 per D (1M)	A1, A16, A8	E2, E3
Zheng (2018)^[63]^	86 (43/43)	60.4 ± 5.2/60.5 ± 5.7	T1, T2, T5, T8/T1, T2, T8	1 per D (20D)	A47, A22, A13, A42, A48, A20, A16, A17, A49, A50, A1, A18, A2	E1, E4
Zhou (2005)^[64]^	51 (30/21)	46–65/45–61	T1, T2, T4/T1, T2, T8	1 per D/3 per D (15D)	A1, A8, A16, A24, A15, A55	E2, E3, E5, E10
Zhuang (2005)^[65]^	40 (20/20)	43–68/45–70	T4/T9	1 per D/3 per D (10D)	A16, A18, A1, A13, A21, A22, A8, A17	E2

### 3.3. Assessment of quality and bias

**Figure 2.**
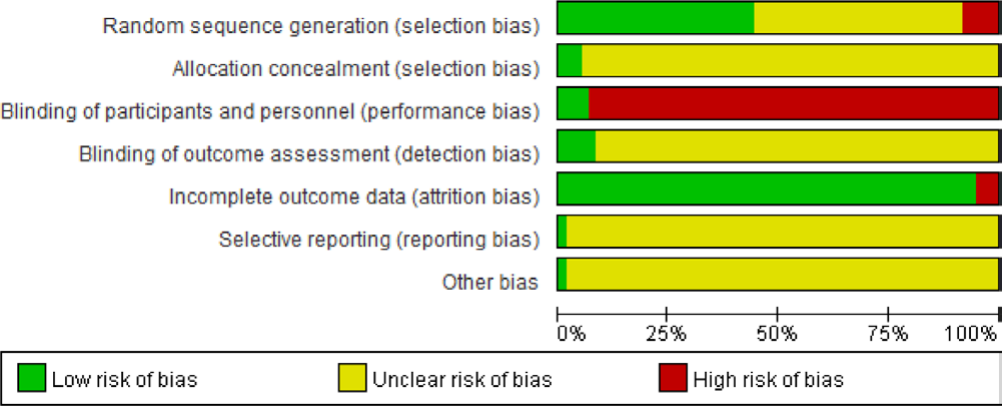
Risk of bias graph: reviewers’ judgements about each risk of bias item presented as percentages across all included studies.

We assessed the methodological quality of the 59 RCTs by using the Cochrane risk-of-bias tool, including sequence generation, allocation concealment, blinding, incomplete data, selective outcome reporting, and other biases. Due to the nature of acupoint therapies, double-blinding of the treatment process was difficult. Data analysis was performed independently in the studies, while the others showed a high risk of bias for data analysis. Five^[31,33,34,59,62]^ studies were double-blinded or single-blinded. A total of 26^[9,13,16–18,21,22,24,28–30,32–34,45–48,51,52,56,57,59–61,65]^ studies reported the method of randomization, which showed a low risk of bias. A summary of the bias is shown in Figure 2, and the risk of bias for each included study is shown in the forest map.

### 3.4. Meta-analysis

**Figure 3.**
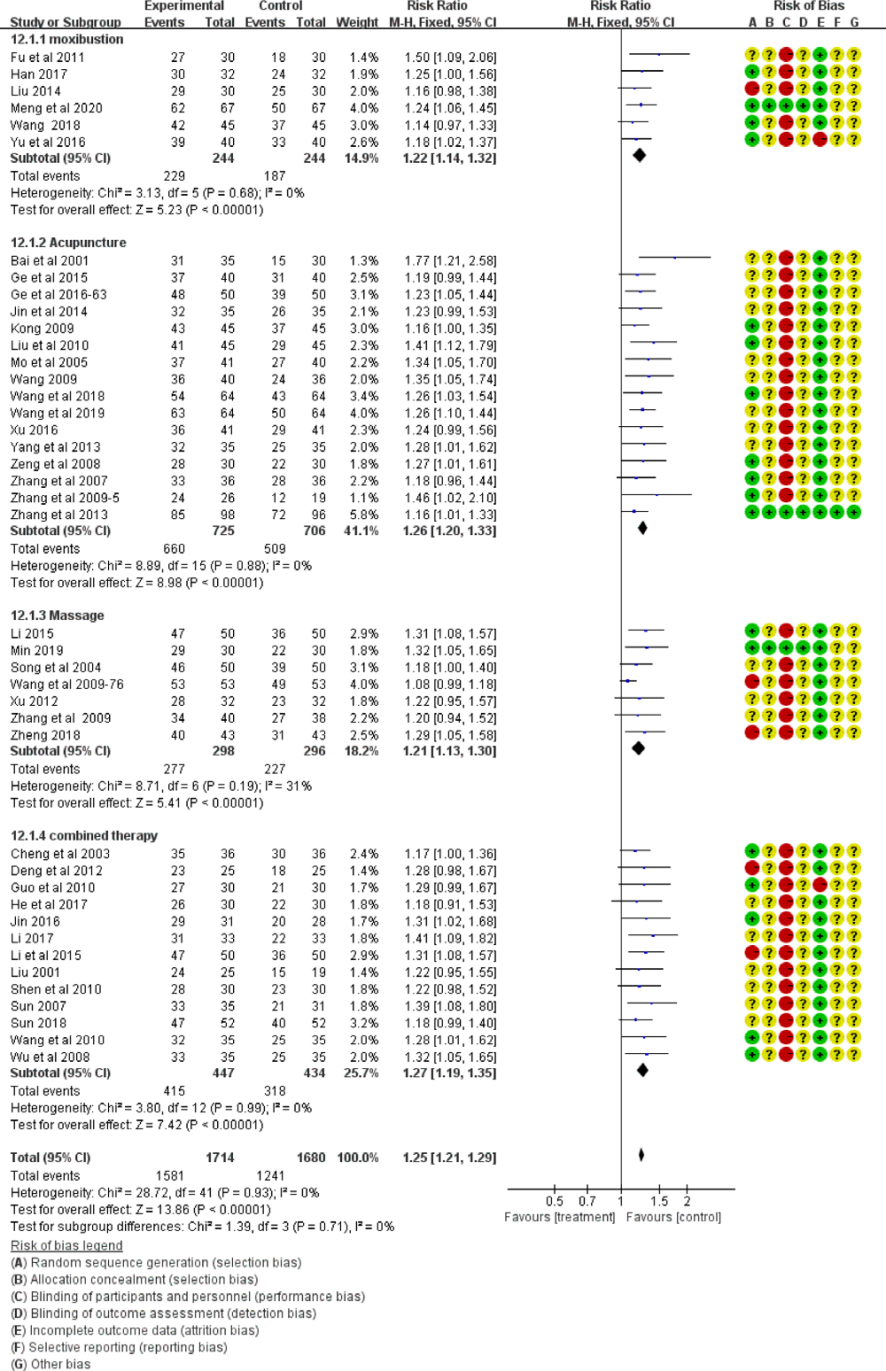
The total effective rate of the traditional Chinese acupoint therapies versus other treatment based on indicators of western medicine. CI = confidence interval, M-H = mantel-haensze.

**Figure 4.**
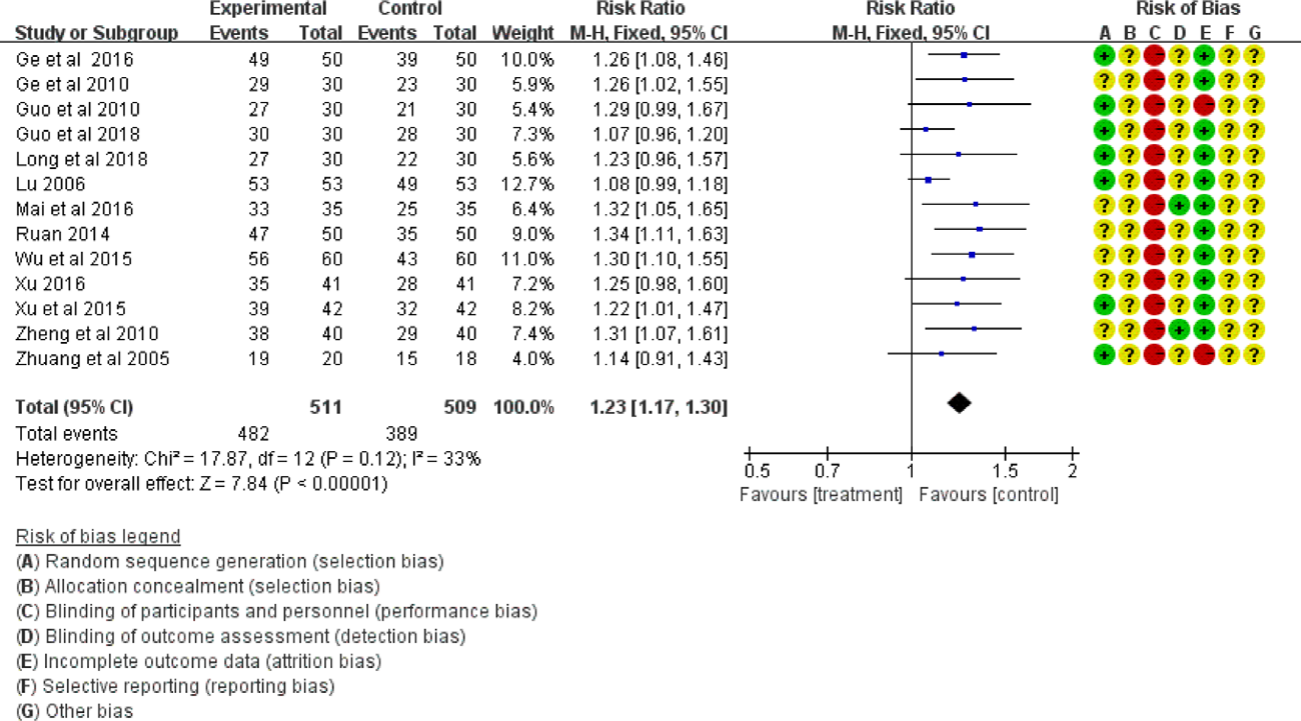
The total effective rate of the traditional Chinese acupoint therapies versus other treatment based on TCM symptoms. CI = confidence interval, TCM = Traditional Chinese Medicine.

**Figure 5.**
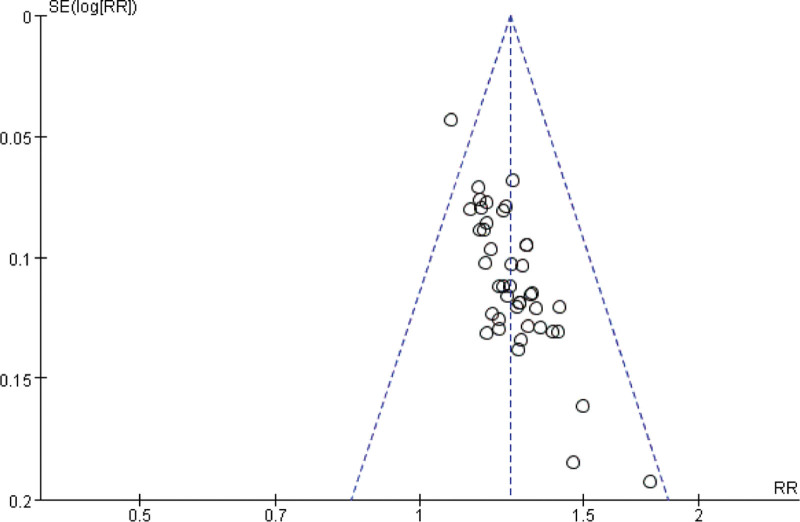
The funnel plot of the traditional Chinese acupoint therapies versus other treatment based on indicators of western medicine. RR = relative risk.

**Figure 6.**
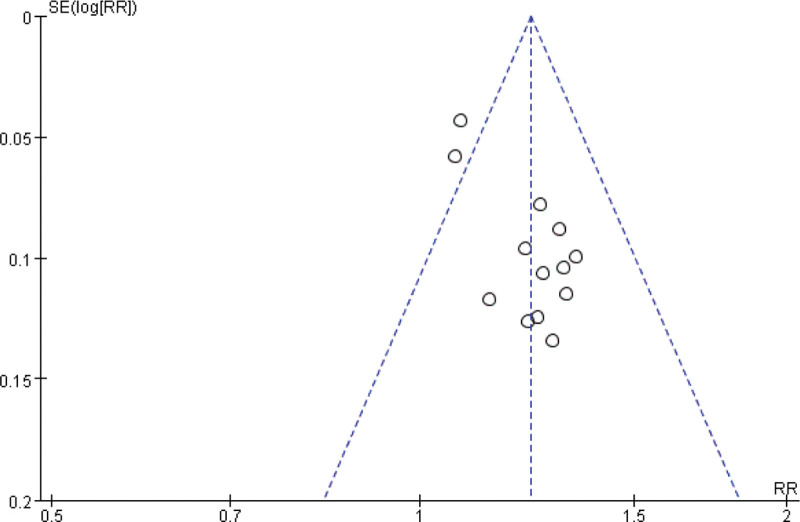
The funnel plot of the traditional Chinese acupoint therapies versus other treatment based on TCM symptoms. RR = relative risk, TCM = Traditional Chinese Medicine.

The total effective rate was analyzed in 53 studies including 4373 participants using indicators of Western medicine or TCM symptoms, of which 53 studies used indicators of both.^[53]^ Forty-two studies based on indicators of Western medicine showed no heterogeneity (χ^2^ = 28.72, df = 41, *P* = .93, *I*^2^ = 0%) using the fixed-effect model (RR = 1.25; 95% CI = 1.21, 1.29; *P* < .00001; see Fig. 3), and 13 studies based on TCM symptoms showed no heterogeneity (χ^2^ = 17.87, df = 12; *P* = .12; *I*^2^ = 33%) using the fixed-effect model (RR = 1.23; 95% CI = 1.17, 1.30; *P* < .00001; see Fig. 4). The difference was statistically significant, indicating that traditional Chinese acupoint therapy treatment was more effective than the control treatments for diabetic gastroparesis. The funnel plot of the publication bias analysis showed that the studies were evenly scattered on both sides of the vertical line. Therefore, there was no significant publication bias in the included articles (Figs. 5 and 6). We conducted a subgroup analysis of 42 studies based on indicators of Western medicine according to different acupoint therapies. The results showed that the massage therapy group had acceptable heterogeneity (χ^2^ = 8.71, df = 6; *P* = .19; *I*^2^ = 31%), while the acupuncture, moxibustion, and combined therapy groups showed homogeneity, and all acupoint therapy groups were more effective than the control groups (see Fig. 3).

**Figure 7.**
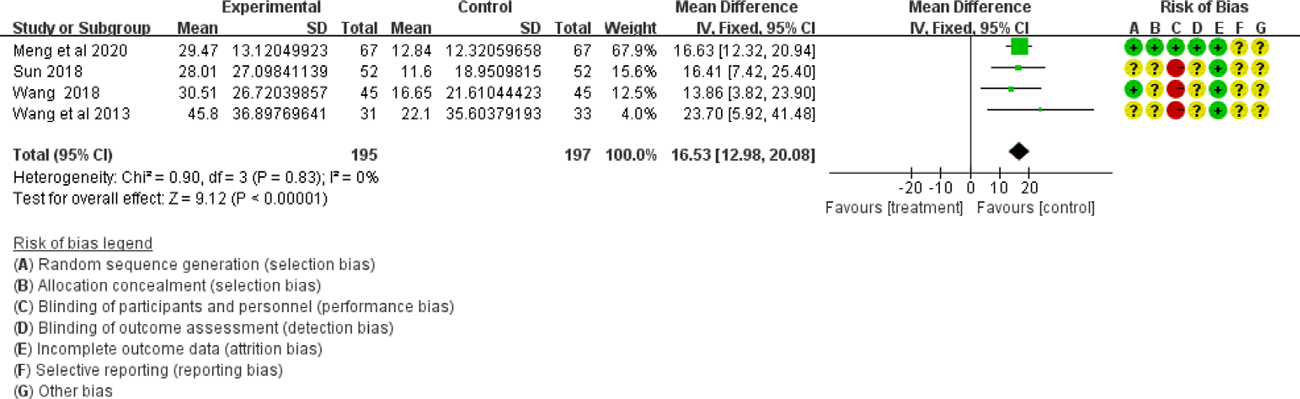
The gastric emptying function of the traditional Chinese acupoint therapies versus other treatment based on gastric emptying rate. CI = confidence interval.

**Figure 8.**
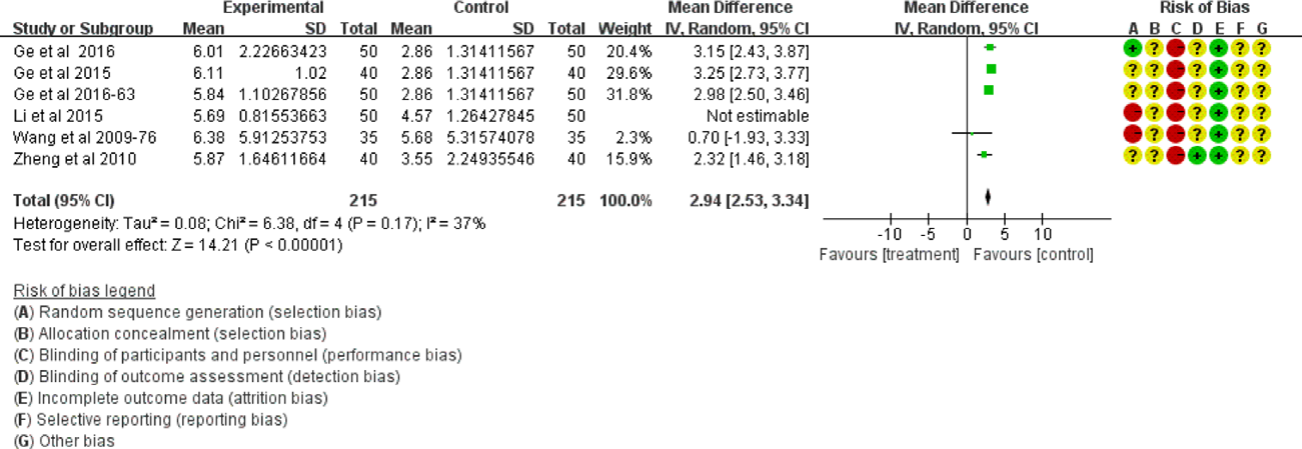
The gastric emptying function of the traditional Chinese acupoint therapies versus other treatment based on gastric barium bar content. CI = confidence interval.

**Figure 9.**
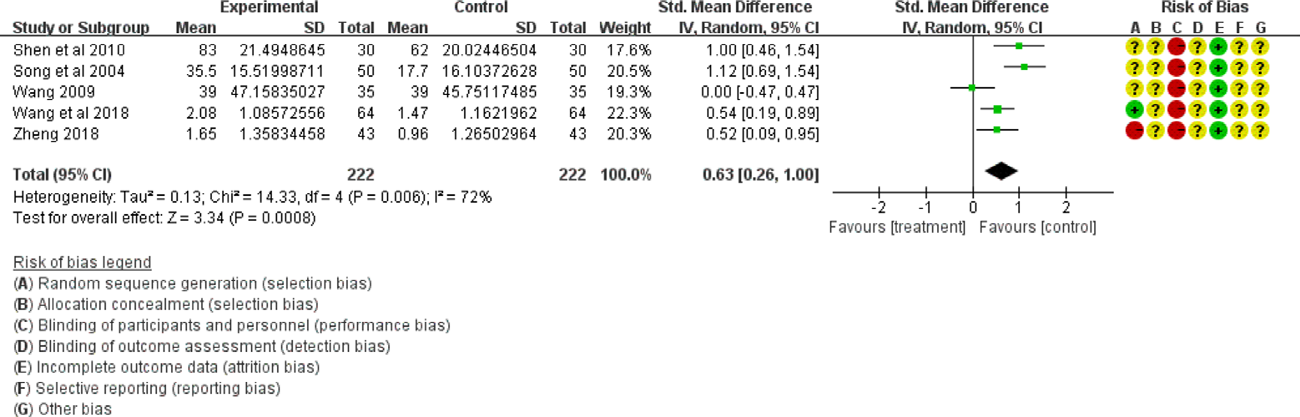
The gastric emptying function of the traditional Chinese acupoint therapies versus other treatment based on gastric emptying time. CI = confidence interval.

Gastric-emptying function was analyzed in 15 studies including 1368 participants. Four studies based on the gastric-emptying rate showed no heterogeneity among the studies (χ^2^ = 0.9; df = 3; *P* = .83; *I*^2^ = 0%) using the fixed-effect model (RR = 16.53; 95% CI = 12.98, 20.08; *P* < .00001; see Fig. 7). However, 6 studies based on gastric barium bar content and 5 studies based on gastric-emptying time showed heterogeneity. Therefore, the literature exclusion method was used for sensitivity analysis. After the exclusion of 1 low-quality study,^[25]^ the results for gastric barium bar content were homogeneous (χ^2^ = 6.38; df = 4; *P* = .17; *I*^2^ = 37%; see Fig. 8), while the results for gastric-emptying time were still heterogeneous (χ^2^ = 14.33; df = 4; *P* = .006; *I*^2^ = 72%) using the random effect model (RR = 0.63; 95% CI = 0.26, 1.00; *P* < .00001; see Fig. 9). All the results showed that the traditional Chinese acupoint therapy treatment group was more effective in improving gastric-emptying function than the control group in diabetic gastroparesis.

**Figure 10.**
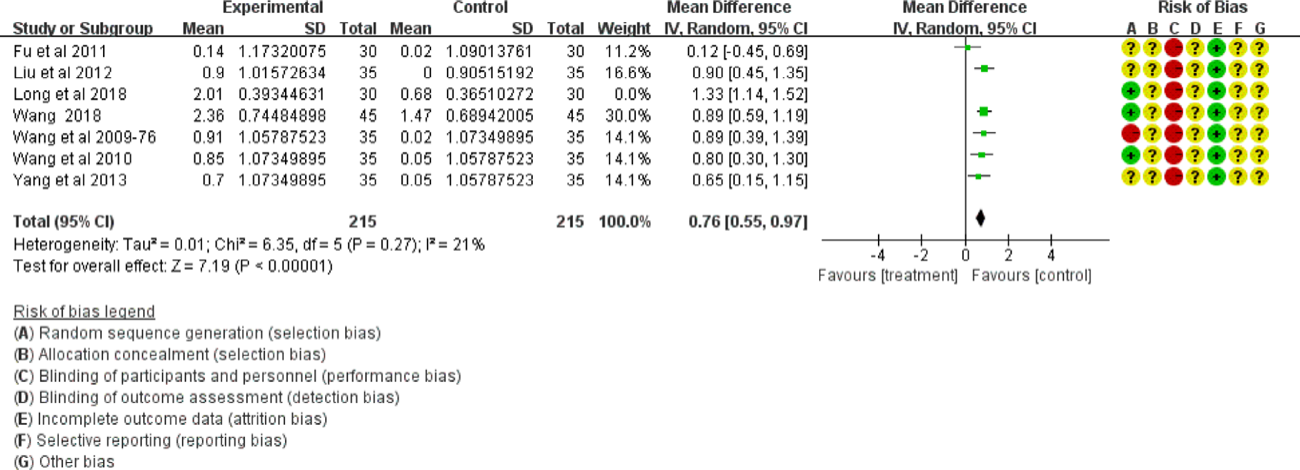
The glycosylated hemoglobin of the traditional Chinese acupoint therapies versus other treatment based on gastric emptying time. CI = confidence interval.

Thirty-one studies analyzed the effect of traditional Chinese acupoint therapy versus other treatments on blood sugar levels in patients with diabetic gastroparesis. Among these studies, 16 studies including 1268 participants analyzed fasting glucose levels, 9 studies including 792 participants analyzed 2-hour blood glucose level, and 7 studies including 490 participants analyzed glycosylated hemoglobin level. However, the results showed heterogeneity, and literature exclusion and subgroup analyses were used to analyze sensitivity. After the exclusion of 1 low-quality study,^[29]^ the results of glycosylated hemoglobin were homogeneous (χ^2^ = 6.35; df = 5; *P* = .27; *I*^2^ = 21%; see Fig. 10), while the other 2 items were still heterogeneous. Finally, we discarded these comparisons.

Six studies including a total of 620 participants analyzed gastrointestinal hormones. Among these, 4 studies including 400 participants analyzed gastrin; all 6 studies analyzed motilin; and 1 study including 66 participants analyzed somatostatin. We discarded these comparisons because of heterogeneity between studies, which could not be eliminated after heterogeneity analysis using the literature exclusion method.

## 4. Discussion

Diabetic gastroparesis is a common complication of diabetes mellitus and mainly manifests as loss of appetite, belching, nausea, vomiting, abdominal distension, abdominal pain, dyspepsia, and burning sensation in the stomach. Treatment methods include controlling blood sugar levels, changing diet habits and structure, and administration of gastric-motility drugs. However, adherence to long-term treatment is difficult because of the side effects of drugs, and the effect is always unsatisfactory.^[67]^

Acupoint therapies are traditional Chinese treatments based on the theory of meridians and acupoints, which regulate the Qi and blood of the viscera to treat diseases.^[68]^ Acupoint therapies appeared earlier than TCM decoctions and have been used for thousands of years with remarkable effects in China. Diabetic gastroparesis belongs to the category of painful abdominal mass. Its pathogenesis involves Yin deficiency, which leads to excess Yang, causing Qi and blood deficiency, qi stagnation, and blood stasis. Acupoints on the spleen and stomach meridians are always used to treat this disease. These acupoints improve gastric contractions, accelerate gastric peristalsis, and promote gastric emptying. A total of 56 acupoints were identified in this study, of which the 15 acupoints listed in Table 2 were used in 5 or more of the included studies (see Table 2). Treatments on Zhongwan, Zusanli, Sanyinjiao, Pishu, Guanyuan, Tianshu, and Weishu can directly regulate gastrointestinal function; Neiguan and Gongsun are always used together to adjust gastrointestinal function; treatments on Taichong have the effect of dredging liver qi, which is beneficial for gastrointestinal function; and treatments on Qihai, Taixi, Shenshu, and Shenque can tonify kidney qi to warm the kidney yang and tonify spleen soil, thereby regulating stomach function. All acupoints are commonly used to gastrointestinal dysfunction.

**Table 2:** Acupoints used in 5 or more of the included studies.

Acupoints	Studies
Zhongwan	45
Zusanli	43
Neiguan	28
Sanyinjiao	17
Pishu	14
Tianshu	14
Guanyuan	11
Weishu	11
Shenque	9
Gongsun	9
Taichong	8
Qihai	7
Taixi	6
Shenshu	6
Quchi	5

In 2018, Kim et al^[69]^ found that acupuncture can significantly improve the symptoms of diabetic gastroparesis patients through systematic review. But in TCM, acupuncture is a small part of acupoint therapy. The acupoint therapy refers to the treatment of diseases by stimulating specific points on the meridians of the body through acupuncture, massage, pressing, moxibustion, etc. So in this meta-analysis, we included studies on single or combined use of acupuncture, moxibustion, and massage and excluded studies using a combination of acupoint therapies and exclude internal administration of TCM drugs. Since chiropractic, auricular acupoint therapy, and acupoint pressing are all manual acupoint treatments, we classified these therapies as massage therapy. Considering the differences in operation and effects of acupuncture, moxibustion, and massage, subgroup analysis based on the total effective rate was conducted to study the heterogeneity and efficacy of different therapies. The subgroup analysis results showed homogeneity of different acupoint therapies (see Fig. 3), and the effective rate was significantly higher than that in the control group. In the subsequent analysis, we combined the results of different therapies, including the total effective rate, indicators of gastric emptying function, blood glucose, and gastrointestinal hormones. As shown in the meta-analysis results above, in comparison with the control groups, the acupoint therapies were associated with an increased total effective rate, enhanced gastric-emptying function, and decreased HbA1c levels. However, a comparative analysis of fasting blood glucose, 2-hour blood glucose, and gastrointestinal hormones could not be performed due to the irreducible heterogeneity between the 2 groups. These results are consistent with a study of self-acupoint massage on blood glucose levels in older adults with type 2 diabetes mellitus, which showed that there were no significant changes in FBG and 2-hour postprandial blood glucose levels between the massage and control groups, while there was a significant difference in HbA1c levels.^[70]^

Bleeding at the stimulated site during acupuncture therapy and slight contusion of the skin tissue during massage therapy are common adverse reactions. However, only 2 of the 59 studies reported adverse effects. In one of the studies,^[49]^ the adverse reaction rate in the treatment group was 3.13%, which was lower than the 15.63% in the control group. Another study showed no obvious adverse reactions in the treatment group and 33.3% in the control group.^[9]^ Considering the low quality of the included studies and the non-negligible publication bias, for example, the possibility of selective data reporting by the authors, we were cautious about the adverse reaction rate. In addition, blinding was difficult to implement due to the nature of acupoint therapy interventions. Moreover, the inclusion of different acupoint therapies may result in significant heterogeneity, and the studies included were only in English and Chinese, which were potential limitations in the meta-analysis.

## 5. Conclusions

In general, the meta-analysis results showed that traditional Chinese acupoint therapies, massage, acupuncture, moxibustion, or acupoint therapies combined with Western medicine and conventional care offered significant advantages in the treatment of diabetic gastroparesis in comparison with Western medicine or conventional care. Acupoint therapies increase the total effective rate, enhance gastric emptying function, and decrease HbA1c levels. In addition, acupoint therapies are minimally invasive, have few side effects, and can be used as complementary therapies for diabetic gastroparesis. However, evidence from high-quality studies is still required.

## Author contributions

XL designed this study. ZY, YS, PG, YF, XC, and JX performed the data extraction and analysis and wrote the initial draft. XW assisted with the data interpretation.

**Data curation:** Xiaoming Li, Zongbao Yan, Jin Xia, Yanan Sun, Peijun Gong, Yuncui Fan, Xiaodong Wang, Xinjie Cui.

**Formal analysis:** Xinjie Cui.

**Funding acquisition:** Xiaoming Li.

**Investigation:** Peijun Gong.

**Writing – original draft:** Xiaoming Li.

**Writing – review & editing:** Xinjie Cui.
